# Structural Insights into Neutrophilic Migration Revealed by the Crystal Structure of the Chemokine Receptor CXCR2 in Complex with the First PDZ Domain of NHERF1

**DOI:** 10.1371/journal.pone.0076219

**Published:** 2013-10-02

**Authors:** Guorong Lu, Yanning Wu, Yuanyuan Jiang, Shuo Wang, Yuning Hou, Xiaoqing Guan, Joseph Brunzelle, Nualpun Sirinupong, Shijie Sheng, Chunying Li, Zhe Yang

**Affiliations:** 1 Department of Biochemistry and Molecular Biology, Wayne State University School of Medicine, Detroit, Michigan, United States of America; 2 Advance Photon Source, Argonne National Laboratory, Argonne, Illinois, United States of America; 3 Nutraceuticals and Functional Food Research and Development Center, Prince of Songkla University, Hat-Yai, Songkhla, Thailand; 4 Department of Pathology, Wayne State University School of Medicine, Detroit, Michigan, United States of America; Saint Louis University, United States of America

## Abstract

Neutrophil plays an essential role in host defense against infection, but uncontrolled neutrophilic infiltration can cause inflammation and severe epithelial damage. We recently showed that CXCR2 formed a signaling complex with NHERF1 and PLC-2, and that the formation of this complex was required for intracellular calcium mobilization and neutrophilic transepithelial migration. To uncover the structural basis of the complex formation, we report here the crystal structure of the NHERF1 PDZ1 domain in complex with the C-terminal sequence of CXCR2 at 1.16 Å resolution. The structure reveals that the CXCR2 peptide binds to PDZ1 in an extended conformation with the last four residues making specific side chain interactions. Remarkably, comparison of the structure to previously studied PDZ1 domains has allowed the identification of PDZ1 ligand-specific interactions and the mechanisms that govern PDZ1 target selection diversities. In addition, we show that CXCR2 can bind both NHERF1 PDZ1 and PDZ2 in pulldown experiments, consistent with the observation that the peptide binding pockets of these two PDZ domains are highly structurally conserved. The results of this study therefore provide structural basis for the CXCR2-mediated neutrophilic migration and could have important clinical applications in the prevention and treatment of numerous neutrophil-dependent inflammatory disorders.

## Introduction

Interleukin 8 receptor, beta (CXCR2) is a G-protein-coupled receptor that mediates neutrophil migration to sites of inflammation and controls the positioning of oligodendrocyte precursors in developing spinal cord by arresting their migration [1,2]. This receptor also functions in angiogenesis and wound healing, and plays an important role in both spontaneous and inflammation-driven tumorigenesis [1,3,4]. In almost all the cases, the ability of CXCR2 to direct cell trafficking and positioning depends on its ability to bind to a repertoire of structurally and functionally related chemokines [1]. For example, CXCR2 can bind all seven ELR-positive CXC chemokines, which include growth-related protein (Gro)-α, -β, and -γ, epithelial-derived neutrophil attractant-78 (ENA-78), granulocyte chemotactic protein-2 (GCP-2), interleukin-8 (IL-8) and neutrophil-activating peptide-2 (NAP-2) [5]. When binding to one of these chemokines, CXCR2 is capable of initiating G-protein heterotrimeric dissociation, which in turn induces many downstream signaling events such as intracellular calcium mobilization and actin polymerization both required for the chemokine gradient-directed cell migration [1].

Although the general process of the CXCR2-mediated signaling is well established, the mechanisms regarding specific coupling of CXCR2 to its downstream signaling molecules still remain poorly understood. We recently showed that CXCR2 formed a complex with its downstream effector phospholipase C (PLC)-β2 via the scaffold protein Na^+^/H^+^ exchanger regulatory factor-1 (NHERF1) in neutrophil-like cell lines and bone marrow-derived neutrophils [6]. We also showed that this complex played a critical role in the CXCR2-mediated signaling and was required for intracellular calcium mobilization and neutrophilic transepithelial migration [6]. Furthermore, we showed that the formation of this complex was mediated by the PDZ domains of NHERF1, which bridged CXCR2 and PLC-β2 by binding to their C-terminal PDZ-binding motifs [6]. Remarkably, the PDZ-mediated interaction of NHERF1 with the C-terminal sequence STTL of CXCR2 was essential for the functional assembly of the CXCR2/NHERF1/PLC-β2 complex, and disrupting the interaction with a cell permeable PDZ motif-containing peptide was sufficient to block the IL-8-induced CXCR2 neutrophilic signaling [6]. As neutrophil dysregulation is central to human immunopathology [7], the identification of this novel CXCR2 complex that contributed to neutrophil chemotactic regulation suggested that targeting this trimeric complex inside the neutrophils might represent a new strategy for the treatment of numerous neutrophil-dependent inflammatory disorders [6]. This notion, in turn, highlights the importance of elucidating the structural basis of the PDZ domain-mediated CXCR2-NHERF1 interaction, as a necessary prerequisite of discovering small molecules that could fine-tune CXCR2 activity or suppress excessive, disease-causing neutrophilic infiltration.

In general, PDZ domains mediate protein interactions by recognizing short amino acid motifs at the C-termini of target proteins, through which PDZ domains play important roles in signal complex assembling and receptor recycling as well as in establishing cell polarity and directing protein trafficking [8]. Recent studies showed that individual PDZ motifs are capable of recognizing up to seven C-terminal ligand residues, with a vast potential to interact with a large number of biologically and functionally diverse ligands [9]. However, in many cases, the specificity of the PDZ-peptide interaction is determined mainly by the residues at positions 0 and -2 of the peptide (position 0 referring to the C-terminal residue), whereas other residues do not significantly contribute to the interaction [10]. Based on that, PDZ domains have been grouped into two major classes. Class I domains bind to peptides with the consensus sequence (S/T)X(V/I/L) (X denoting any amino acid), while class II domains recognize the motif (F/Y)X(F/V/A)[11,12]. Corroborating this classification, structural studies revealed that PDZ domains, including NHERF1 PDZ1 [12,13], adopt a conserved overall fold characterized by six strands (1–6) and two -helices (A and B) [10,11]. They also revealed that PDZ domains share a similar peptide recognition mode, with the 0 residue of peptide occupying a hydrophobic pocket and the -2 residue participating in direct side chain interactions [10,11].

In fact, the structural similarity in PDZ ligand recognition [10,11], together with the fact that more than 250 PDZ domains in over 150 different proteins are present in the human genome [14] has led to years of intensive research regarding how PDZ domains, a structurally simple protein interaction module, can achieve effective ligand discrimination, the nature of which, however, still remains obscure. In this context, it is interesting to note that PDZ binding is also enormously promiscuous, with one domain capable of binding multiple targets [15]. For example, NHERF1 contains two PDZ domains (PDZ1 and PDZ2) that are known to interact with a variety of transmembrane proteins, such as the cystic fibrosis transmembrane conductance regulator (CFTR), the 2-adrenergic receptor (2AR), the platelet-derived growth factor receptor (PDGFR) and the parathyroid hormone receptor (PTHR) [16,17]. Moreover, PDZ promiscuity is exemplified by the fact that some PDZ domains have the ability to bind peptide sequences that belong to both class I and class II motifs [18]. Therefore, these examples have made it apparent that detailed analysis and comparison of many proteins will be required to establish and illuminate the full range of ligand discrimination operated by the PDZ domain fold [19]. A high-resolution structural interpretation of individual PDZ domain function should in turn provide considerable insights into the mechanisms whereby ligand specificity and promiscuity dictate the diversification of biological functions. For this reason, we report here the high-resolution structure (1.16 Å) of the NHERF1 PDZ1 domain in complex with the CXCR2 C-terminal peptide TSTTL. The structure reveals PDZ1 ligand-specific interactions and the mechanisms that govern the PDZ1 target selection diversity. We also show that CXCR2 can bind both NHERF1 PDZ1 and PDZ2 in pulldown experiments, consistent with the observation that the two domains share highly structurally-conserved peptide binding pockets. The results of this study therefore provide important insights into the CXCR2-mediated neutrophilic migration and could be valuable in the development of novel therapeutic strategies against many neutrophil-dependent inflammatory disorders.

## Results and Discussion

### Structure Determination

To facilitate NHERF1-CXCR2 cocrystallization and reveal the mechanism by which NHERF1 recognizes CXCR2, we generated a chimeric protein with the C-terminus of the NHERF1 PDZ1 domain (residues 11–94) fused to five amino acids (TSTTL) corresponding to the CXCR2 residues 356–360. We reasoned that such design would take advantage of functional interaction between CXCR2 and NHERF1, allowing efficient crystal packing by promoting intermolecular contacts in a more site-specific manner. This strategy has previously been applied to NHERF1 PDZ1 and several other PDZ-target complexes [12,13,20], and indeed proved to be effective in obtaining diffraction-quality PDZ1-CXCR2 crystals in this study. The crystals diffracted to high resolution (1.16 Å), and the structure was determined by molecular replacement. The model was refined to R_work_ of 18.6% and R_free_ of 20.8%, and the evaluation of its stereochemistry using PROCHECK showed that 91.9% of the residues are in the most favored, 8.1% in the additional allowed, and 0.0% in the generously allowed regions; no residues is found in the disallowed regions (Table 1).

**Table 1:** Crystallographic data and refinement statistics.

Space group	*P*3_1_21
Cell parameters (Å)	
a=b	50.4
c	66.0
Wavelength (Å)	0.97872
Resolution (Å)	20.0-1.16 (1.20-1.16)
*R*_*merge*_^a^	0.063 (0.463)^b^
Redundancy	9.7 (7.0)
Unique reflections	33912
Completeness (%)	100 (100)
I/	19.1 (3.3)
**Refinement**	
Resolution (Å)	20.0-1.16
Molecules/AU	1
*R*_*work*_^c^	0.186 (0.217)
*R*_*free*_^d^	0.208 (0.248)
RMSD	
Bond lengths (Å)	0.011
Bond angels ()	1.2
No. of atoms	
Protein	655
Peptide	36
Water	102
Chloride	3
B-factor (Å^2^)	
Protein	20.4
Peptide	15.1
Water	27.8
Chloride	16.4

a *R*_*merge*_=Σ|I-I|/ΣI, where I is the observed intensity and I is the averaged intensity of multiple observations of symmetry-related reflections.

b Numbers in parentheses refer to the highest resolution shell.

c *R*_*work*_= Σ|F_o_-F_c_|/Σ|F_o_|, where F_o_ is the observed structure factor, F_c_ is the calculated struture factor.

d *R*_*free*_ was calculated using a subset (5%) of the reflection not used in the refinement.

### Overview of the Structure

**Figure 1:**
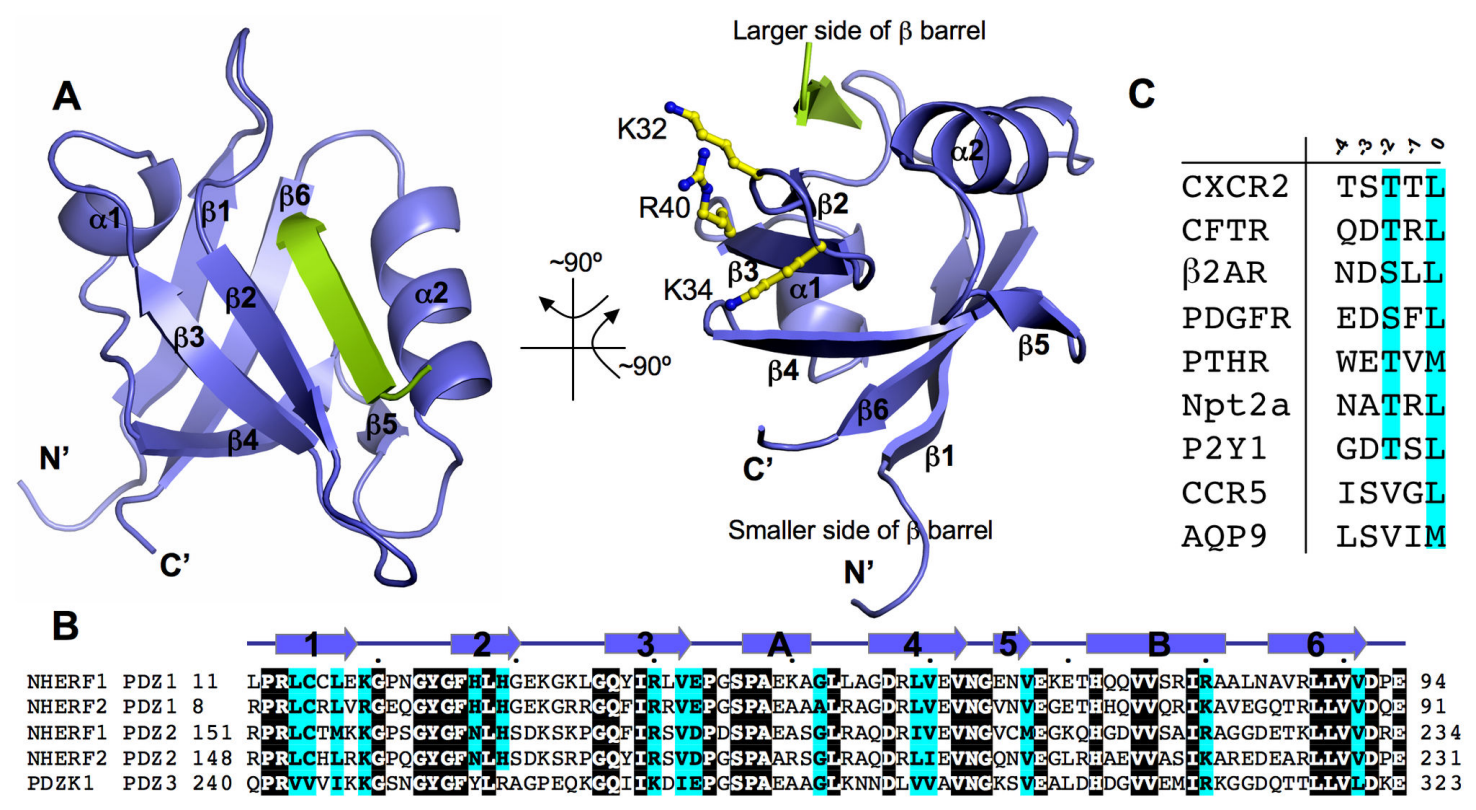
Structure of NHERF1 PDZ1 in complex with the CXCR2 C-terminal sequence TSTTL. (A) Ribbon diagram of the PDZ1-CXCR2 structure, front view on the left and side view on the right. PDZ1 is shown in purple and the CXCR2 peptide shown in green. Secondary structures of PDZ1, α-helices and β-strands, are labeled and numbered according to their position in the sequence. Side chains of putative PDZ1 lipid-binding residues are depicted by balls-and-sticks in the side view of the structure. (B) Sequence alignment of selected PDZ domains. The alignment was performed by ClustalW [45], including human NHERF1, human NHERF2 and mouse PDZK1. Identical residues are shown as white on black, and similar residues appear shaded in cyan. Secondary structure elements are displayed above the sequences and labeled according to the scheme in Figure 1A. Sequence numbering is displayed to the left of the sequences, with every 10th residue marked by a dot shown above the alignment. (C) Sequence alignment of the last five residues of natural NHERF binding targets. The alignment includes CXCR2, CFTR, 2AR, PDGFR, PTHR, Npt2a (type 2 sodium-phosphate cotransporter), purinergic receptor P2Y1, CCR5 (C-C chemokine receptor type 5), and AQP9 (aquaporin 9). Protein names are shown at the left of the sequences. Position numbering is displayed above the alignment, with position 0 referring to the very C-terminal residue.

The crystal structure reveals a polymeric PDZ1 arrangement with the carboxyl terminal region TSTTL of one PDZ1 molecule bound to a neighboring PDZ1, which leads to the formation of a linear PDZ1 filament throughout the crystals. The overall topology of NHERF1 PDZ1 is similar to other PDZ domains [11], consisting of a six-stranded -barrel (1–6) that is capped top and bottom by two -helices (A and B) (Figure 1A). The -barrel has a hydrophobic interior, lined up with highly conserved residues, including Leu59, Ile39, Phe26, Cys15, Val86, Leu88, Leu59, and Val90 (Figure 1B). These residues are also evolutionally conserved across other PDZ motifs, suggesting their universal role in stabilizing PDZ fold by forming a continuous hydrophobic core [10]. In contrast, the outside of the barrel is rather hydrophilic, with a region enriched with basic residues predicted to be involved in membrane association and direct interaction with acidic lipids [21]. These putative lipid-binding residues include Lys32, Lys34 and Arg40, which are located within 3 and its preceding loop with their side chains facing toward the solvent (Figure 1A). The interaction of cholesterol with these surface residues was required for dynamic NHERF1-CFTR colocalization, and disruption of the NHERF1’s cholesterol-binding activity resulted in aberrant CFTR channel activation [21].

**Figure 2:**
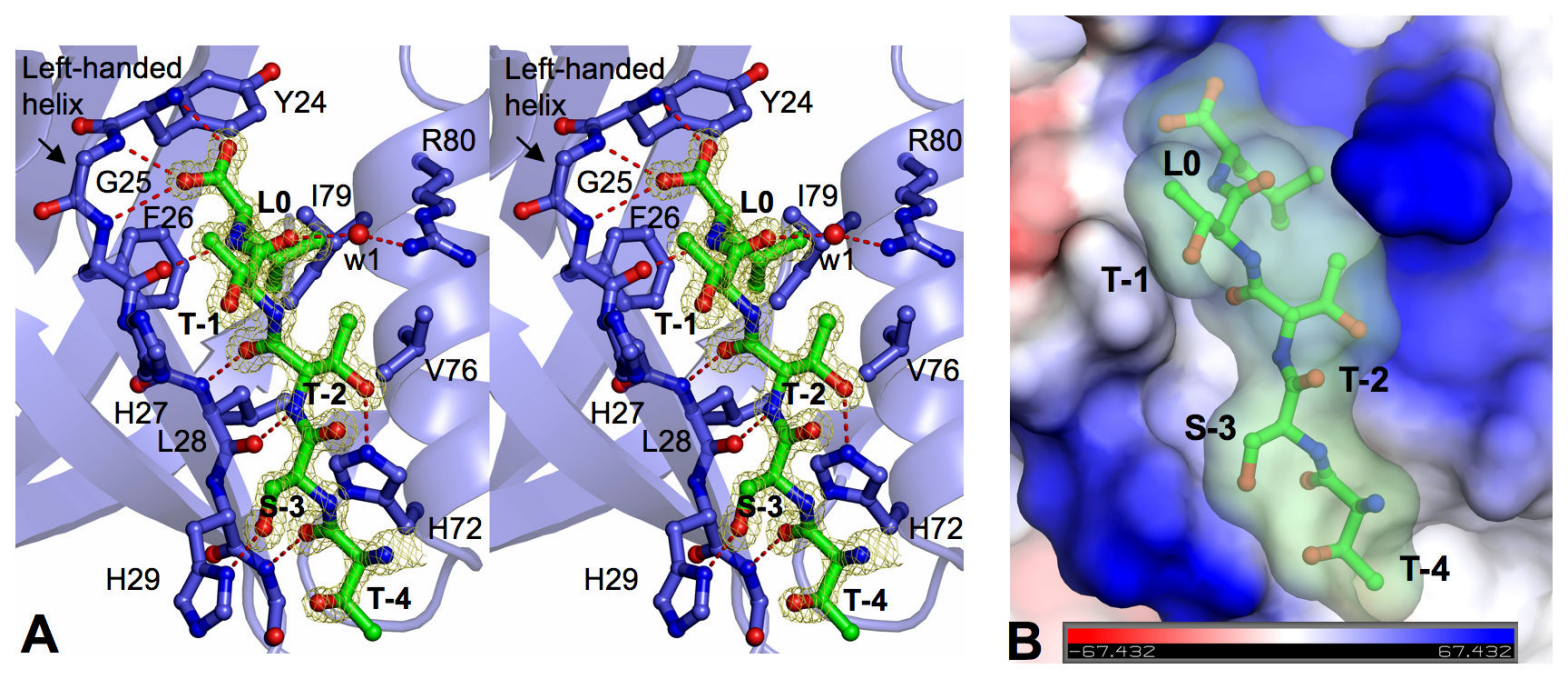
Interactions between PDZ1 and CXCR2. (A) Stereo view of the PDZ1 ligand-binding site bound to the CXCR2 C-terminal peptide. PDZ1 residues are represented by balls-and-sticks with their carbon atoms colored in purple. CXCR2 peptide is depicted by balls-and-sticks overlaid with 2Fo − Fc omit map calculated at 1.16 Å and contoured at 1.8 σ. Hydrogen bonds are illustrated as red broken lines. (B) Surface representation of the PDZ1 binding pocket with coloring according to the electrostatic potential: red, white, and blue correspond to negative, neutral and positive potential, respectively. The vacuum electrostatics/protein contact potential was generated by PyMOL. The CXCR2 peptide is depicted by balls-and-sticks overlaid by its transparent molecular surface.

In addition to its amphipathic nature, the PDZ1 -barrel is structurally asymmetric, having a circular cross section that is larger at one end than the other (Figure 1A). At the smaller end, the PDZ1 N- and C-termini curl close together and block the barrel opening. On the opposite end, a helix (B) is positioned in a manner that still permits access to the barrel’s interior core region. This helix (B) is stabilized by VDW contacts with the residues from β3 and 4 but stays ~9 Å apart from β2. The nearly parallel arrangement of B and β2 creates a shallow surface groove approximately 18 Å long, 8 Å wide, and 4 Å deep. The groove stretches deeply into the central cavity of the -barrel, forming a peptide-binding pocket that is responsible for highly robust protein interactions [9]. Similar to other PDZ structures [12,13], the CXCR2 C-terminal peptide TSTTL inserts into the PDZ1 binding pocket as an additional -strand antiparallel to 2 (Figure 2). In this setting, the invading peptide is highly ordered as indicated by high quality electron density maps (Figure 2A) and below average B factors (Table 1). It should be noted that the CXCR2-binding pocket is topologically distinct from the putative lipid binding sites (Figure 1B), and that mutation of the cholesterol-binding residues did not lead to significant changes in the NHERF1 ligand-binding activity [21]. Although the role of cholesterol in CXCR2 signaling is currently unknown, the PDZ topological asymmetry that places the CXCR2-binding sites opposite to the domain termini, along with direct cholesterol-NHERF interaction being important for cell signaling and protein networking [21], suggests a signaling platform with PDZ1 serving as a dual-specificity scaffold to bring together the membrane and juxtamembrane signaling complexes [21].

### Specificity Determinants of Consensus PDZ1 Binding Motif

The CXCR2 pentapeptide (TSTTL) binds PDZ1 in an extended conformation, forming numerous contacts with β2 and B and burying a total solvent-accessible surface area of 692 Å^2^ (Figure 2). Only the last four residues of CXCR2 make specific contacts to PDZ1, whereas the first threonine adopts a well-defined conformation but is not directly involved in PDZ1 recognition. This indicates that this residue may not contribute to the interaction specificity, consistent with recent finding that the residue at the corresponding peptide position does not have any contacts with PDZ1 in the solution structure of the PDZ1/synthetic CFTR peptide complex [22]. Similar to other PDZ domains [10], the specificity and affinity of the PDZ1-CXCR2 interaction are achieved mainly by the residues at positions 0 and -2 of the peptide (position 0 referring to the very C-terminal residue), whereas residues -1 and -3 appear to be important for ligand-specific interactions (see below). Specifically, the side chain of CXCR2 Leu0 enters a deep hydrophobic pocket composed of invariant residues Tyr24, Phe26, and Leu28 from β2, and Val76 and Ile79 from B. These pocket-forming residues are important for NHERF1 functions; for example, mutation of Tyr24 and Phe26 completely abolished the NHERF1-targets interaction and significantly altered cellular processes essential to tumor metastatic behaviors [23].

In the PDZ1 pocket, the position of Leu0 is fully secured by a hydrogen bond from its carbonyl oxygen to the Tyr24 amide nitrogen and by triple hydrogen bonding to the PDZ1 carboxylate-binding motif (Figure 2A). The carboxylate-binding motif, located between β1 and β2, has a left-handed helical conformation that results in three amide nitrogens being directed toward the peptide, thereby allowing the hydrogen-bond formation between the Leu0 carboxylate and the amides of Phe24, Gly25, and Phe26. In addition, Leu0 fits tightly in the PDZ1 pocket, with the side chain directly abutting the benzene ring of Phe26 and the isobutyl group of Ile79. Remarkably, the surface of the pocket is highly complementary to the shape of leucine (Figure 2B), which thus provides a structural basis that governs the high affinity binding between CXCR2 and NHERF1 [6]. This stereochemical complementarity also suggests that any model that substitutes Leu0 to larger hydrophobic residues would generate substantial steric clashes; to smaller ones would be energetically unfavorable. Interestingly, recent molecular dynamic simulation studies showed that replacement of Leu0 by Val or Ala of the CFTR ligand resulted in fewer interactions with NHERF1 PDZ1 and substantially lower binding energy [24]. Collectively, the present structure demonstrates the PDZ1 binding selectivity for the CXCR2 C-terminal leucine, which is mediated by the stereochemically complementary hydrophobic interaction in a fashion that is highly conserved in class I PDZ motif [10]. This conserved binding selectivity in turn provides structural rationalization for the importance of Leu0 in CXCR2 function. The competition experiments using the leucine-mutated peptides did not affect IL-8-induced CXCR2 signaling, but the treatment of bone marrow neutrophils with a CXCR2 peptide containing an intact PDZ motif, disrupting NHERF1-CXCR2 complex, resulted in a significant inhibition of intracellular calcium mobilization, chemotaxis, and transepithelial migration of neutrophils [6].

Another conserved feature of the PDZ1-CXCR2 interaction is that Thr-2 engages in numerous specific contacts with PDZ1 and plays an important role in determining the specificity and affinity of the interaction. Specifically, the amide nitrogen of Thr-2 hydrogen bonds to the carbonyl oxygen of Leu28, while the backbone carbonyl of Thr-2 hydrogen bonds to the main chain amide of the same residue. In addition, the side chain hydroxyl of Thr-2 hydrogen bonds with the imidazole ring of His72, with its side chain aliphatic carbon making direct hydrophobic contacts to the conserved Val76. These observed interactions are consistent with biochemical studies showing that direct contacts between ligand -2 residue and the residues from PDZ B helix are critical for the binding specificity of class I PDZ-ligand interaction [9,10]. For example, mutation of the His72-equivalent residue in ERBB2IP-1 to Tyr, Asn, Gln or Lys, all capable of forming hydrogen bonds to threonine, did not alter specificity significantly, whereas substitution of the residue with Leu, Val or Met resulted in class II specificity profiles with preference for hydrophobic residues at -2 position [9]. Therefore, our structure, coupled with these previous results, indicates that the stabilization and specificity of PDZ1-CXCR2 interaction are dependent on both Leu0 and Thr-2 that possess the ability to form networks of hydrogen bonds and hydrophobic interactions with NHERF1.

### Ligand-specific PDZ1-CXCR2 interactions

Compared to the motif residues (0 and -2), the peptide residues at positions -1 and -3 are largely exposed, with both side chains oriented upwards in the PDZ1-CXCR2 complex (Figure 2). As a result of this orientation, the -1 and -3 residues make fewer direct contacts with PDZ1 and bury a much less extent of solvent-accessible surface area than the motif residues (87 Å^2^, Thr-1; 84 Å^2^, Ser-3; 127 Å^2^, Leu0; 103 Å^2^, Thr-2). These findings are consistent with previous evidence that both -1 and -3 residues in the peptide ligands were less stringently specified by individual PDZ domains than the residues at the 0 and -2 positions [10]. Specifically, the interactions with Thr-1 include a direct polar contact from its side chain hydroxyl to the side chain of His27 and a water-mediated hydrogen bond between its carbonyl oxygen and the side chain of Arg80 (Figure 2A). In these aspects, the PDZ1-CXCR2 structure differs significantly from the structures of other PDZ1-ligand complexes. In PDZ1-CFTR, the guanido group of Arg-1 forms two salt bridges to the Glu43 side chain and two hydrogen bonds with the carbonyl oxygen of Asn22 [12], while in PDZ1-2AR and PDZ1-PDGFR, the nonpolar residues at position -1 of the peptide ligands engage in direct hydrophobic interactions with the imidazole ring of His27 [13]. These observed differences reveal that there is considerable diversity in PDZ1 interaction with -1 residue of different ligands, manifested by four chemically different amino acids (Asn22, His27, Glu43, and Arg80) combined in the discrete ways to discriminate the ligand residues of different hydrophobicity and polarity. We speculate that this diversity may reflect a high degree of selectivity in NHERF1 ligand recognition, consistent with a vast potential for PDZ domain family to bind different sequences [9].

The interactions between PDZ1 and CXCR2 at position -3 of the peptide are also very different from other PDZ1 complexes. In PDZ1-CXCR2, the hydroxyl group of Ser-3 forms a direct hydrogen bond with the His29 side chain (Figure 2A), whereas the side chain of residue Asp-3, which is common in CFTR, 2AR, and PDGFR, is engaged in salt bridge interaction with the Arg40 guanidinium and direct hydrogen bonding to the His27 side chain [12,13]. We speculate that these structural differences may be important for PDZ1 ligand discrimination, as it was shown that highly specific contacts with different types of contextual residues contributed significantly to the binding specificities of all peptide-mediated protein interactions [25]. In agreement with this conclusion, the structure of the NHERF2 PDZ2 in complex with the PSTRL sequence revealed the occurrence of similar interactions between Ser-3 and a histidine residue (His166) of the PDZ domain [20]. Remarkably, the NHERF2 PDZ2 His166 residue corresponds to NHERF1 PDZ1 His29 (Figure 1A), suggesting that the amino acid at this position may play a critical role in specific ligand recognition via interaction with the -3 residue of the peptide. Taken together, the present structure indicates that the peptide residues at positions -1 and -3 contribute to ligand specific PDZ1-CXCR2 interactions, suggesting that these positions may have been naturally selected to facilitate PDZ ligand selection within a complex network of NHERF-scaffolded interactions [9]. Interestingly, the residues at the -1 and -3 positions exhibit significant variability across natural NHERF1 binding targets, with the two-residue combination unique to each characterized ligand (Figure 1C).

The considerable contacts between PDZ1 and the residues at positions -1 and -3 suggest that these residues may play an important role in the affinity of the PDZ1-CXCR2 interaction. Consistent with this suggestion, affinity selection experiments showed that NHERF PDZ1 almost exclusively selected ligands with arginine at position -1 from random peptides, and mutation of Arg to Ala, Phe, Leu, or Glu decreased the affinity of the PDZ1-ligand interaction by 2-10 fold [12,26]. In addition, it has been shown that position -3 is also an important determinant of binding affinity, with PSD-95 preferring to bind peptides with acidic side chains at this position [10]. Furthermore, analysis of the binding specificities for nearly half of over 330 PDZ domains in human and worm revealed that there was a strong correlation between natural PDZ sequences and ligand specificities at both -1 and -3 positions of peptides [9]. Remarkably, the PDZ binding preferences at these positions can be influenced by multiple structural and chemical mechanisms involving both direct contacts and cooperative, long-range effects, suggesting that binding specificities can evolve rapidly, thus enabling PDZ for robust differentiation between biologically diverse ligands [9]. Therefore, our structure, together with these previous findings, suggests that the ligand specific contacts between PDZ1 and the CXCR2 -1 and -3 residues are important for the binding affinity and specificity of the PDZ1-CXCR2 interaction. In a broad term, the ligand specific interactions at these positions could lead to different PDZ-ligand complex stabilities, which, in conjunctions with an increasingly complex NHERF interaction network [27], may determine signaling orchestration and underlie the highly coordinated regulation of manifold NHERF-controlled signaling events [28]. In support of this idea, recent biochemical studies suggested that NHERF1, NHERF2, and CAL competed to regulate CFTR endocytic processing, and the differences in their CFTR binding affinities were required for CFTR to efficiently escape CAL-mediated degradation through repeated rounds of uptake and recycling [16].

### Structural Comparison Reveals PDZ1 Target Selection Specificity

**Figure 3:**
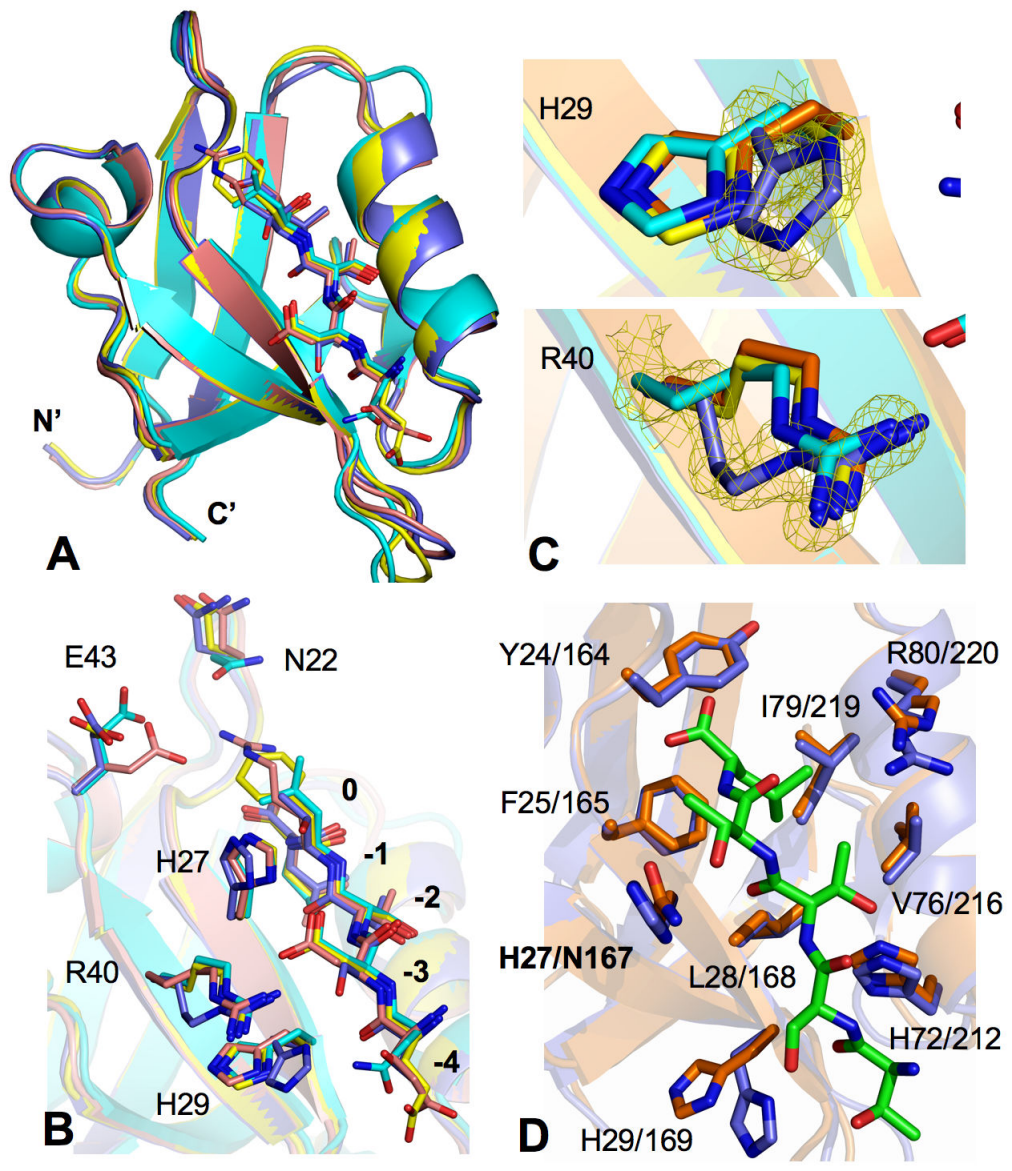
Structural comparison of PDZ domains. (A) Superposition of the structures of PDZ1-CXCR2 (purple; PDB code: 4JL7), PDZ1-CFTR (orange; PDB code: 1I92) [12], PDZ1-2AR (cyan; PDB code: 1GQ4) [13], and PDZ1-PDGFR (yellow; PDB code: 1GQ5) [13]. PDZ domains are represented by ribbon, while residues in the ligands are displayed as sticks. (B) Superposition of the PDZ1 ligand binding pockets. Both PDZ1 and ligand residues are depicted by sticks and colored according to the scheme in Figure 3A. (C) Close-up views of structural differences of His29 (top) and Arg40 (bottom). The CXCR2 peptide is depicted by sticks overlaid with 2Fo − Fc omit map calculated at 1.16 Å and contoured at 2.0 σ. (D) Superposition of NHERF1 PDZ1 (purple) and PDZ2 (pink; PDB code: 2OZF) peptide binding pockets. CXCR2 peptide is shown in green and PDZ residues are depicted by balls-and-sticks.

To uncover the structural details that govern the CXCR2-NHERF1 ligand specific interactions, we compared the PDZ1-CXCR2 structure to the crystal structures of all available NHERF1 PDZ1-ligand complexes, including PDZ1-CFTR, PDZ1-2AR, and PDZ1-PDGFR [12,13]. The structural comparison reveals that the four PDZ1 structures are highly similar, with pairwise RMSDs (root-mean-square differences) for entire Cα atoms ranging from 0.33 to 0.64 Å (Figure 3A). Larger differences in the PDZ1 backbone are found at two loop regions (2-3 and B-6 loops), but note that these loops made of non-conserved residues (Figure 1B) are conformationally flexible, as indicated by poorly defined electron density and higher than average B factors (data not shown). Moreover, the backbone conformations of the bound peptides are also highly superimposed (RMSDs from 0.09 to 0.15 Å), as are their relative spatial positions to the conserved PDZ1 motifs (Figure 3A). These findings therefore indicate that the binding of different peptides has little effect on the PDZ1 overall fold, consistent with previous studies showing that the localized changes at a few key positions within the PDZ fold were responsible for dramatically altered PDZ binding specificity [29]. Indeed, significant differences are observed only in the peptide-binding pocket, especially at PDZ residues that are involved in recognition of different side chains at position -1 and -3 of the peptide ligands. In particular, the structural alignments reveal that the Asn22 side chain has two different orientations, while the conformation of the Glu43 side chain differs among all four PDZ1 structures (Figure 3B). Such structural differences have been noted before and led to the conclusion that the conformational changes of Asn22 and Glu43 underlay the PDZ1 flexibility to accommodate ligands with -1 side chains of different hydrophobicity and polarity [13].

The availability of the PDZ1-CXCR2 structure, however, not just confirms the above conclusion, but also has the potential to reveal differential -3 side chain recognition, i.e., how PDZ1 differentiates CXCR2 Ser-3 from Asp-3 of CFTR, 2AR, and PDGFR. In this context, it is interesting to note that the most striking difference among the PDZ1 complexes is the His29 side chain, which adopts two different conformations. In PDZ1-CXCR2, the side chain of His29 is oriented toward the hydroxyl group of Ser-3, participating in specific ligand interaction; whereas in other three complexes, the imidazole ring of His29 points away from the bound ligands and does not engage in any peptide recognition (Figure 3B). Strikingly, this conformational change is accompanied by large alteration in the Arg40 rotameric state, which rotates to make completely different PDZ1-peptide interactions. In PDZ1-CFTR, PDZ1-2AR, and PDZ1-PDGFR, Arg40 is a key anchor residue for specific Asp-3 recognition and participates in direct ligand binding [12,13]. In PDZ1-CXCR2, due to steric effects, the reorientation of His29 forces the Arg40 side chain to kink outwards and prevents it from interacting with the shorter side chain of Ser-3 (Figure 3B). Therefore, these observed differences demonstrate that the structural variability surrounding the peptide-binding pocket is important for PDZ1 ligand specific interactions, and that the rotameric differences of a few key residues constitute the basis for PDZ1 robustness to bind a diverse array of functionally different proteins [9,29].

### CXCR2 Interacts with Both NHERF1 PDZ1 and PDZ2

**Figure 4:**
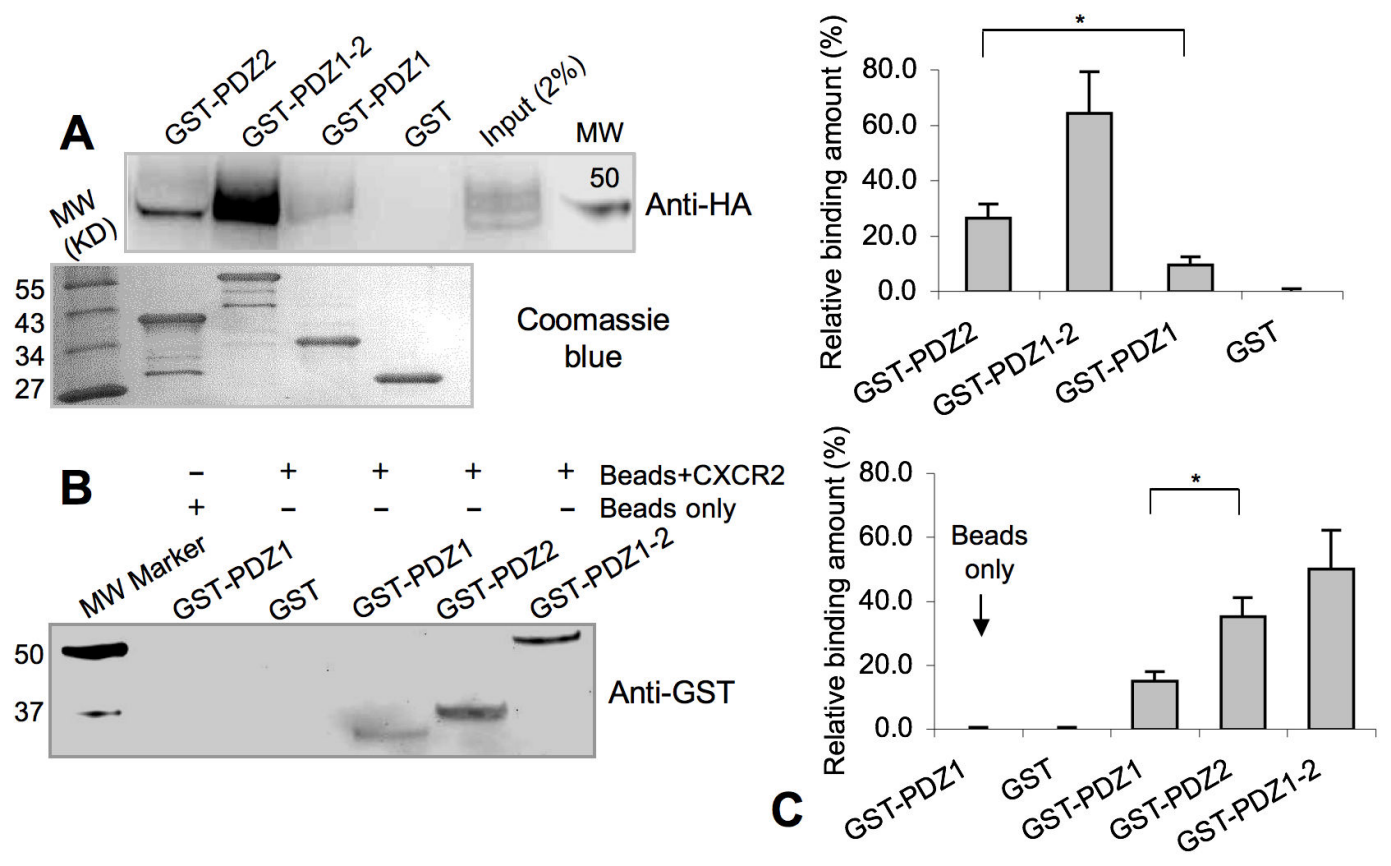
CXCR2 interacts with both PDZ1 and PDZ2 of NHERF1. (A) GST pull-down of CXCR2 with NHERF1. Lysates of HEK293 cells overexpressing HA-tagged CXCR2 were used as prey. GST fusion proteins of NHERF1 PDZ1, PDZ2, and PDZ1-PDZ2 were used as bait. GST alone served as a negative control. Binding experiments were analyzed by SDS-PAGE and visualized by immunoblot using anti-HA antibodies. The amount of beads-immobilized GST proteins in each reaction is shown in the lower panel. (B) Biotin pull-down assays to detect direct interaction between CXCR2 and NHERF1. A biotinylated peptide corresponding to the last 13 residues of CXCR2 was used as bait, while purified GST-PDZ1, GST-PDZ2, GST-PDZ1-PDZ2 and GST alone as prey. Binding was resolved by SDS-PAGE and immunoblotted with anti-GST antibodies. (C) All experiments performed in (A) and (B) were repeated three times. The results were quantified using the CCD gel imager (UVP Chemidoc) and presented as mean±standard deviation. The asterisks indicate statistically significant differences (P < 0.05) between the values indicated by the brackets. Statistical analysis was performed using the two-tailed Student’s t-test. Top: GST pull-down of CXCR2 with NHERF1, and bottom: biotin pull-down of NHERF1 PDZ domains with the CXCR2 peptide.

The structural alignment reveals that NHERF1 PDZ1 and PDZ2 share highly similar overall structures and also highly conserved ligand binding pockets (Figure 3C). The only notable difference in the ligand binding sites is residue 27, which is His in PDZ1 and Asn (residue 164) in PDZ2. It should be noted that this conserved substitution maintains the amino functionality of the side chains, and thus, is not expected to disrupt the observed polar interactions between the CXCR2 peptide and PDZ1 (Figure 2A). Based on that, we hypothesize that NHERF1 PDZ2 may also bind to CXCR2. Indeed, we showed that CXCR2 interacts with both PDZ1 and PDZ2 in the GST-pulldown experiments, with PDZ2 exhibiting higher binding affinities (Figure 4). Specifically, we overexpressed CXCR2 in HEK293 cells and then performed pulldown assays from cell lysates using various GST-PDZ constructs. Whereas no CXCR2 was detected in the control lane containing GST alone, significant amounts of CXCR2 were found in the lanes containing PDZ1 domain (GST-PDZ1), PDZ2 domain (GST-PDZ2), and both PDZ domains together (GST-PDZ1-PDZ2) (Figure 4A and 4C). To test whether the PDZ-CXCR2 interactions are direct, we performed in vitro pulldown experiments with a biotinylated peptide corresponding to the last 13 amino acids of CXCR2. Similar binding results were observed in the experiments where CXCR2 interacts with both PDZ domains of NHERF1 (Figure 4B and 4C).

Many other NHERF1 ligands, such as CFTR, PDGFR, and PTH1R, were also known to bind both PDZ1 and PDZ2 in vitro [16,30,31], but in most cases, the biological significance of such bivalent interactions remains unknown. It has been shown that bivalent binding was important for CFTR channel gating regulation, and disruption of the PDZ2-CFTR interaction, but keeping the interaction between PDZ1 and CFTR intact, was able to abolish the NHERF1 stimulatory effect on CFTR channel open probability [32,33]. In addition, it has been suggested that a single NHERF1 molecule could assemble a PDGFR dimer and played a role in PDGFR signaling via stabilizing the ligand-induced receptor dimerization [34]. Later studies, however, revealed that PDGFR signaling was unexpectedly enhanced rather than impaired in NHERF1-null mouse embryonic fibroblasts, suggesting quite a different role of this bivalent molecule in PDGFR signaling regulation [35]. Remarkably, a recent article by Cardone et al. showed that NHERF1 PDZ1 and PDZ2 domains differently regulated invadopodia and podosome dynamics [23], and suggested that the differential functions of two PDZ domains might be dependent on their ability to interact with a unique array of functionally different signaling molecules [23]. Therefore, it is reasonable to speculate that the ability of CXCR2 to bind both NHERF1 PDZ domains may allow CXCR2 to operate in different signaling networks, which might be a key functional trait that has evolved to deal with the complexity of signaling transduction. While the biological impacts of this bivalent binding are currently unknown, future studies should be directed toward evaluation of its effects on CXCR2-mediated neutrophilic migration, receptor dimerization, CXCR2 internalization, and especially determining whether different NHERF1 PDZ domains could mediate the assembly of distinct and specific CXCR2 signal transduction complexes.

### Drug Design Perspective

Due to the exceptional importance of CXCR2 in inflammation and tumorigenesis [4], the structural determinants of the CXCR2-NHERF1 interaction may be valuable in developing new methods and strategies for targeted drug discovery. For example, this information can be used to create new CXCR2 inhibitors that are potent and specific to block the CXCR2-NHERF1 interaction. Such inhibitors could in turn have a therapeutic potential in inhibiting neutrophil-driven inflammation by reducing neutrophil recruitment and restoring neutrophils to the tissue clearance pathway of apoptosis [36]. In this context, it is interesting to note that disruption of the CXCR2-NHERF1 complex was sufficient to inhibit the IL-8-induced neutrophilic chemotaxis and margination [6]. Therefore, small molecules and peptides that specifically block the CXCR2-NHERF1 interaction could act as CXCR2 antagonists and could be useful in attenuating the signaling activities of CXCR2 in various neutrophil-related inflammation disorders, such as inflammatory bowel diseases, chronic lung inflammation, and atherosclerosis [6].

However, the commonality of peptide recognition at position 0 and -2 by class I PDZ domains, together with NHERF1 capable of binding to a multitude of ligands (Figure 1C), poses a challenge for designing CXCR2 inhibitors that are specific to the CXCR2-NHERF1 interface but do not cross-react with any of the other NHERF1-mediated interactions. NHERF1, through a network of PDZ domain-mediated interactions, regulates many cellular processes essential to normal physiological functions, such as testicular differentiation, signal transduction, endosomal recycling, membrane targeting, and hormone receptor desensitization [37,38]. Therefore, it is conceivable that random targeting of NHERF1-ligand interactions by nonselective inhibitors could disrupt the NHERF1 interaction network and leads to considerable risks with a diverse range of unwanted physiological and hormonal abnormalities. Regarding this possibility, it is particularly important to note that contextual specificity plays a key role in all peptide-mediated protein interactions [25], suggesting that the ability to achieve CXCR2 inhibitor selectivity is dependent on the identification and exploitation of structural features that differentiate CXCR2 from other NHERF1 binding partners, and on understanding how the peptide motif and context work in coordination to control the specificity and formation of each crucial NHERF-scaffolded signaling complex. This notion is consistent with accumulating evidence that the positions other than 0 and -2 make significant and variable contributions to both affinity and specificity of the PDZ-mediated interactions [11,13]. For example, recent large-scale PDZ specificity mapping studies demonstrated that the PDZ domain family is surprisingly complex and diverse, recognizing up to 7 C-terminal ligand residues and forming at least 16 unique specificity classes across human and worm [9]. Furthermore, we recently showed that, despite the motif-contacting residues involved in CXCR2 binding are all conserved in NHERF1 and PDZK1 (Figure 1A), CXCR2 did not interact with PDZK1 in the *in vitro* GST pull-down assays [6], reciprocally suggesting that high affinity CXCR2 binding and selection by NHERF1 is also context dependent. Therefore, strategies aiming at exploiting CXCR2-NHERF1 contextual interactions may represent a promising approach for the development of small molecules that would selectively block this interaction and specifically inhibit the neutrophil-driven inflammation. In this context, it is particularly important that the ligand-specific structural principles that govern the NHERF1 target-selection diversity should be addressed in great detail.

## Materials and Methods

### Protein Expression and Purification

For X-ray crystallography, a DNA fragment encoding the human NHERF1 PDZ1 (residues 11–94) was amplified by PCR using the full-length human NHERF1 cDNA as a template. The C-terminal extension TSTTL that corresponds to residues 356–360 of human CXCR2 was created by inclusion of 15 extra bases in the reverse primer. The PCR products were cloned in the pSUMO vector containing a N-terminal His6-SUMO tag. The resulting clone was transformed into *Escherichia coli* BL21 Condon Plus (DE3) cells for protein expression. The transformants were grown to an OD600 (optical density at 600 nm) of 0.4 at 37 °C in LB medium, and then induced with 0.1 mM isopropylthio-β-D-galactoside and grown an additional 16 h at 15 °C. The cells were harvested by centrifugation and lysed by French Press. The soluble fraction was then subjected to Ni^2+^ affinity chromatography purification, followed by the cleavage of the His6-SUMO tag with yeast SUMO Protease 1. PDZ1 was separated from the cleaved tag by second Ni^2+^ affinity chromatography and further purified by size-exclusion chromatography. Finally, the protein was concentrated to 10–20 mg/ml in a buffer containing 20 mM Tris–HCl (pH 8.0), 150 mM NaCl, 1 mM β-mercaptoethanol (BME), and 5% glycerol. For pulldown experiments, NHERF1 PDZ1, PDZ2, or PDZ1 and PDZ2 together was cloned into the BamHI/XhoI sites of pGEX4T-1 plasmid, and then transformed into the *Escherichia coli* BL21 Gold (DE3) for protein expression. The proteins were expressed essentially similar as described earlier and purified by affinity chromatography using immobilized glutathione Sepharose 4B resin.

### Crystallization, Data Collection and Structure Determination

Crystals were grown by the hanging-drop vapor-diffusion method by mixing the protein (~8 mg/ml) with an equal volume of reservoir solution containing 100 mM sodium acetate, pH 4.6, 2.7 M sodium chloride at 20 °C. Crystals typically appeared overnight and continued to grow to full size in 3-4 days. Before X-ray diffraction data collection, crystals were cryoprotected in a solution containing mother liquor and 25% glycerol and flash cooled in liquid nitrogen. The data were collected at 100 K at beamline 21-ID-F at the Advanced Photon Source (Argonne, IL) and processed and scaled using the program HKL2000 [39]. Crystals belong to space group P3 _1_21 with unit cell dimensions a = b = 50.4 Å, c = 66.0 Å, and one molecule in the asymmetric unit. The structure was solved by the molecular replacement method with program PHASER [40] using the PDZ1-CFTR structure (PDB code: 1I92) as a search model. The structure modeling was carried out in COOT [41], and refinement was performed with BUSTER [42]. To reduce the effects of model bias, iterative-build OMIT maps have been used during model building and structure refinement [43]. The final models were analyzed and validated with PROCHECK [44]. All figures of 3D representations of the PDZ1-CXCR2 structure were made with PyMOL (www.pymol.org).

### Cell Culture and Transfection

HEK293 cells were obtained from the American Type Culture Collection (Manassas, VA) and maintained as described previously [6]. Briefly, the cells were cultured in Dulbecco’s modified Eagle’s medium (DMEM) (Invitrogen) supplemented with 10% fetal bovine serum (FBS), 100 units/ml penicillin, and 100 g/ml streptomycin. The cells were maintained at 37 °C in a 5% CO_2_-95% air atmosphere and routinely passaged at a ratio of 1:4 when 70-80% confluent. Transfection was carried out with the Lipofectamine 2000 (Invitrogen) transfection kit according to the manufacturer’s protocol. HEK293 cells were plated in 75-cm^2^ flasks. After reaching of 80%–90% confluency, cells were provided with 12 ml of fresh medium and transfection was performed with pcDNA3.1 vector encoding HA-tagged human CXCR2.

### Pulldown Assays

GST pulldown assays were preformed essentially similar as described in our previous studies [6]. Briefly, HEK293 cells overexpressing CXCR2 proteins were lysed with cell lysis buffer (PBS, 0.2% Triton X-100) supplemented with a mixture of protease inhibitors (1 mM phenylmethylsulfonyl fluoride, 1 g/ml of aprotinin, 1 g/ml of leupeptin, and 1g/ml of pepstatin) and phosphatase inhibitor mixture (Sigma). The cell lysates were cleared by centrifugation at 16,000 g for 10 min, and then incubated with GST-NHERF1 fusion constructs (GST-PDZ1, GST-PDZ2, GST-PDZ1-PDZ2) or GST alone for 3 h at 4 °C. After incubation, the complex was mixed with glutathione-agarose beads (BD Biosciences) and incubated for 1 h at 4 °C with general shaking. The beads were then washed three times with 1 ml of lysis buffer, pelleted at 500g for 30 s, and boiled in Laemmli sample buffer. Finally, HA-tagged CXCR2 proteins, which bound to GST-NHERF1 proteins, were resolved by SDS-PAGE and detected by anti-HA antibodies. To verify the direct CXCR2/NHERF1 interaction, purified GST-NHERF1 PDZ domains or GST alone were mixed with a synthetic CXCR2 C-tail peptide (last 13 residues with a biotin-conjugate at N-terminus) in binding buffer (PBS, 0.2% Triton X-100, and protease inhibitors) at 20°C for 1 h. The mixtures were incubated with Streptavidin beads (for binding to biotin-conjugate in the peptide) for 2 h. The beads were then washed three times with binding buffer, and eluted with Laemmli sample buffer containing β-mercaptoethonal. The eluents were resolved by SDS-PAGE, and immunoblotted with anti-GST antibodies.

### Protein Data Bank Accession Number

Coordinates and structure factors have been deposited in the Protein Data Bank with accession number 4JL7.
